# The ethnomycological knowledge of Karajá indigenous people from Bananal Island, Brazil

**DOI:** 10.1371/journal.pone.0311716

**Published:** 2024-10-11

**Authors:** Mazulkieliche Jeronimo dos Reis, Lucas Leonardo-Silva, Solange Xavier-Santos

**Affiliations:** Laboratory of Basic and Applied Mycology and Scientific Dissemination (FungiLab), State University of Goiás, Anápolis, Goiás, Brazil; University of Sao Paulo, BRAZIL

## Abstract

The Cerrado is home to a diversity of traditional communities, among which indigenous and quilombola peoples stand out. The Karajá are one of the ethnic groups in this biome, with a rich history and culture that goes back centuries. They mainly inhabit the regions of the Araguaia and Javaés rivers, occupying lands in the states of Goiás, Mato Grosso, Pará and Tocantins. Considering the importance of studies on ethnomycological knowledge in indigenous communities for preserving culture and the environment, especially in relation to fungi, our objective was to investigate the ethnomycological relationships of the Karajá indigenous people who inhabit Bananal Island, located in Tocantins. Data were collected from applying a semi-structured questionnaire and interviews with 140 people who compose the Macaúba (39%), Fontoura (31%) and Santa Isabel do Morro (30%) communities; they had an average age of 33.9 years, and 62% are male. We observed that the Karajá people recognize the fungi of the environment in which they live, showing a clearer perception of typical morphological groups, such as mushrooms and bracket fungi (wood ears). Although fungi are not used as a component of their diet, the Karajá recognize that some species may have this potential. Furthermore, they use fungi as adornments and decorations in festivities in a playful way, and occasionally for medicinal purposes. Therefore, we can state that this ethnic group does not have a total aversion to fungi, being considered partially mycophilic. This study is a pioneer among Cerrado indigenous people, and reinforces the need to expand research to other communities in different regions in order to expand ethnomycological knowledge among different ethnicities. These investigations could contribute to both an appreciation and conservation of the traditions and knowledge of original Brazilian people, as well as the biodiversity in which they are inserted.

## Introduction

Ethnomycology, in a *sensu lato* concept, is the study of traditional knowledge, manifestations and cultural/environmental implications arising from the relationships between human and fungi, across time and space [1]. It can also be seen as the study of the relationship between fungi and humans in traditional communities, local knowledge and the use of these fungi, highlighting their ecological and cultural importance [2].

The interaction between fungi and culture has deep roots in history, with records of this relationship in Africa, Siberia, Europe, Asia and America. Fungi are integral elements of the biological culture of traditional people, forming part of the biocultural heritage of a country [3]. Furthermore, the relationship between people and fungi can be used as an instrument to direct discussions about local biodiversity within a historical-cultural context.

Few ethnomycological studies have been conducted in Brazil, which most focusing on indigenous populations in the Amazon [4–6]. Those carried out in the Northeast region with rural and indigenous communities from the Caatinga are scarce and recent [7–9]. In the Central-West, investigations into ethnomycological knowledge were conducted through quilombola communities [10] and farmers [11] of the Cerrado biome. In the Southeast, research has focused on fungi that can be used as food [12, 13], while in the Southwest, the focus is on the ethnomycological knowledge of rural community [14].

Traditional indigenous communities residing in the national territory total 817.9 thousand people, distributed among 305 ethnicities [15]. The Iny people are one of Brazil’s ethnic groups with a rich history and culture dating back centuries. They mainly inhabit the regions of the Araguaia and Javaés rivers, occupying lands in the states of Goiás, Mato Grosso, Pará and Tocantins [15, 16]. The ethnic group has a population of 4,326 people [17] and composes three subgroups named by themselves according to the position they occupy on the course of the Araguaia: Javaé, Xambioá and Karajá. Each village establishes a specific territory for fishing, hunting and ritual practices, internally demarcating cultural spaces known to the entire group [15].

This ethnic group belongs to the Macro-Jê linguistic trunk, which uses the native language *Iny rybè*, as well as Portuguese. They have a social division in which men play roles such as defending the territory, opening fields, fishing, building houses and political leadership. Women are responsible for raising children, doing household chores, making ceramic dolls, painting for rituals and preparing food for festivals [16, 18].

The ethnic group traditionally subsists on fishing, subsistence agriculture and collecting fruits and roots from the Cerrado. Fishing plays a crucial role in their survival, as fish is a fundamental element in their diet. In addition, they also grow corn, cassava, sweet potatoes and beans; however, part of their diet also currently comes from industrialized products [18]. Art and ceramics are important cultural expressions for these people. They are known for their skill in making decorative pottery, which is used in rituals and ceremonies, as well as for commercial purposes. The pieces are characterized by colorful geometric designs and representations of animals and human figures [16, 18].

Ethnomycological studies play a fundamental role in providing a deep understanding of the relationship between indigenous communities and fungi. These studies contribute not only to the preservation of traditional knowledge related to medicinal properties, food uses, recreational, ritualistic, and other cultural practices associated with fungi, but also to preventing the gradual loss of this knowledge over time. In Brazil, no ethnomycological studies have been conducted among the Karajá communities, although a study on the perception of children and adolescents about fungi in the community has already been published [19]. Considering that studies on ethnoknowledge in indigenous communities can support the preservation of culture and the environment, as well as contribute to Brazilian ethnomycology, our objective was to understand the ethnomycological relationships of the Karajá communities living on Bananal Island.

## Materials and methods

### Study area

Bananal Island, located on the border of the states of Tocantins, Mato Grosso (along the Araguaia River) and Goiás (in the southern part of the Javaés River), is a river island with approximately 20,000 km^2^ in area. Since 1959, it has been recognized as a Brazilian Environmental Reserve. The island is divided between the municipalities of Formoso do Araguaia, Lagoa da Confusão and Pium. A part of the island is occupied by the Parque do Araguaia Indigenous Land, which covers the southern portion and a large part of the western portion of Bananal, extending to the latitude of the municipality of Santa Terezinha (MT) [17, 20, 21].

Bananal Island is mainly occupied by the Karajá and Javaé indigenous people. The former mainly live in villages located in the western part, mostly on the banks of the Araguaia River. In turn, the Javaé occupy the island interior and on the banks of the Javaés River, the smaller branch of the Araguaia River. This study focuses on the Karajá indigenous people, the population living on Bananal Island, from the Fontoura, Macaúba and Santa Isabel do Morro communities.

The area is characterized by the transition strip between the Amazon Forest and the Cerrado, presenting characteristics of a floodplain subject to seasonal flooding [20–22]. The predominant climate on Bananal Island is tropical hot semi-humid (AW), according to the Köppen climate classification, with a maximum temperature of 38°C during the dry period (between the months of August and September) and a minimum temperature of 22°C in the month of July [23].

Cerrado vegetation predominates in the studied communities, considered the second largest biome in Brazil and South America, surpassed only by the Amazon Forest, originally occupying more than two million km^2^ (23% of Brazil’s entire area). It covers the states of Goiás, Mato Grosso, Mato Grosso do Sul, Minas Gerais, Bahia, São Paulo, Ceará, Maranhão, Piauí, Paraná, Rondônia, Tocantins, and the Federal District, in addition to some isolated spots in Roraima, Amapá, Amazonas and Pará [24]. The Cerrado is a tropical savannah in which low vegetation mainly formed by grasses coexists with scattered trees and shrubs [25]. Furthermore, the biome exhibits a great diversity of soils and climates which are reflected by vast biota [24].

### Sampling universe and data collection

The study met the requirements of Resolution 510/2016 of the National Health Council, having been authorized by the following institutions: The Research Ethics Committee (*Comitê de Ética em Pesquisa—CEP*) of the State University of Goiás (UEG), The National Research Ethics Council (*Conselho Nacional de Ética em Pesquisa—CONEP*) (opinion no. 5,693,844/2022), The National Foundation of Indigenous Peoples (*Fundação Nacional dos Povos Indígenas—FUNAI*) (process no. 08620.008900/2022-23) and The National Council for Scientific and Technological Development (*Conselho Nacional de Desenvolvimento Científico e Tecnológico—CNPq*) (process no. 01300.011596/2022-75).

**Fig 1:**
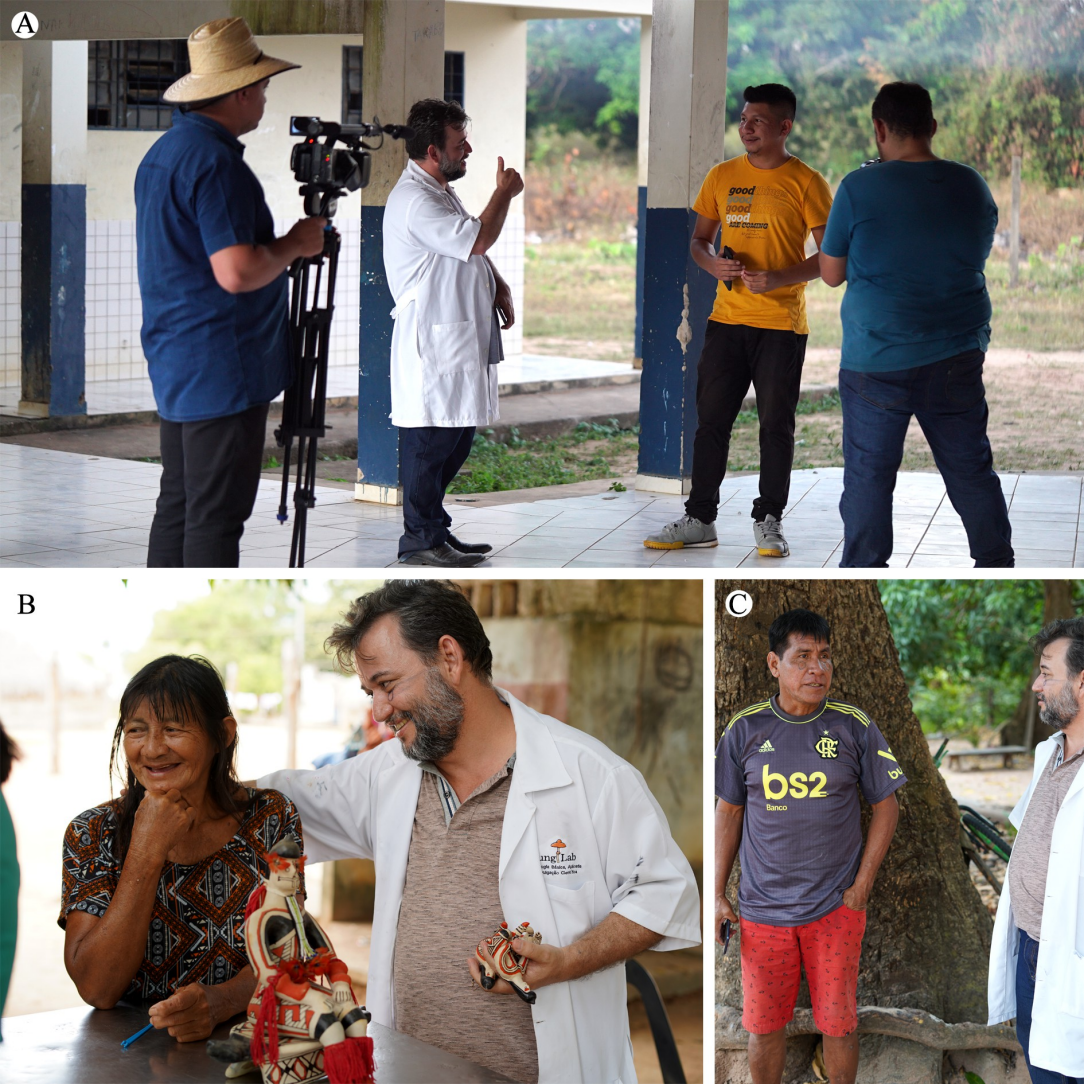
Data collection. (A) Recording of interviews. (B‒C) Interview with community members.

Members of the three studied communities (Fontoura, Macaúba and Santa Isabel do Morro—Iny people of the island) were interviewed, all over the age of 18 and who agreed to participate in the study (Fig 1). The selection of interviewees took place during a meeting with members of each community, organized by the local leaders and guides, where we presented the project and extended the invitation to participate. Upon acceptance, the participants signed an Informed Consent Form (ICF). The interviews were recorded (Fig 1) and the anonymity of the participants was maintained. The interview was conducted in Portuguese, and we had translators when necessary to facilitate communication.

Data collection occurred between January and April 2023, according to Cunha [10] through individual interviews using an image album with 21 specimens of fungi, including myxomycetes, and a semi-structured questionnaire to guide the main topics to be covered and facilitate dialogue (Table 1). The image album used was printed, composed of species common in the region and from different morphological and ecological groups: bracket fungi (wood ears), gasteroid, lichens, molds and mildews, mushrooms (agaricoid), plant (phytopathogen), insects (entomopathogenic) and human (mycoses) pathogens, and myxomycetes. In addition to the questions in the questionnaire, additional information was also recorded when relevant.

**Table 1:** Questionnaire applied to 140 interviewees to assess their ethnomycological knowledge.

**Sociodemographic data**
1. Age: 2. Gender: 3. Marital status: 4. Religion:
**Ethnomycological data**
**1**. Do you recognize any of these organisms from the image album? If so, which ones?
**2**. Do you consider these organisms as: () Plant, () Animal, () Fungi or mushroom, () Other thing, () I don’t know
**3**. How do you name these organisms?
**4**. Since when have you known these organisms? () Children, () Youth, () Adult, () Unknown
**5**. Who/where did you learn about these organisms from? () Family, () Teacher/School, () Nobody, () Other
**6**. What do these organisms need to grow?
**7**. Where can we find these organisms?
**8**. Are any of these organisms important? Why?
**9**. Do you use them or know someone who does?
**10**. Do you have any experience or know any stories about the fungi shown in the image album or others? For example, their use as food, medicine, in rituals, that glows in the dark, and their potential to cause diseases in humans, animals, and plants.

**Fig 2:**
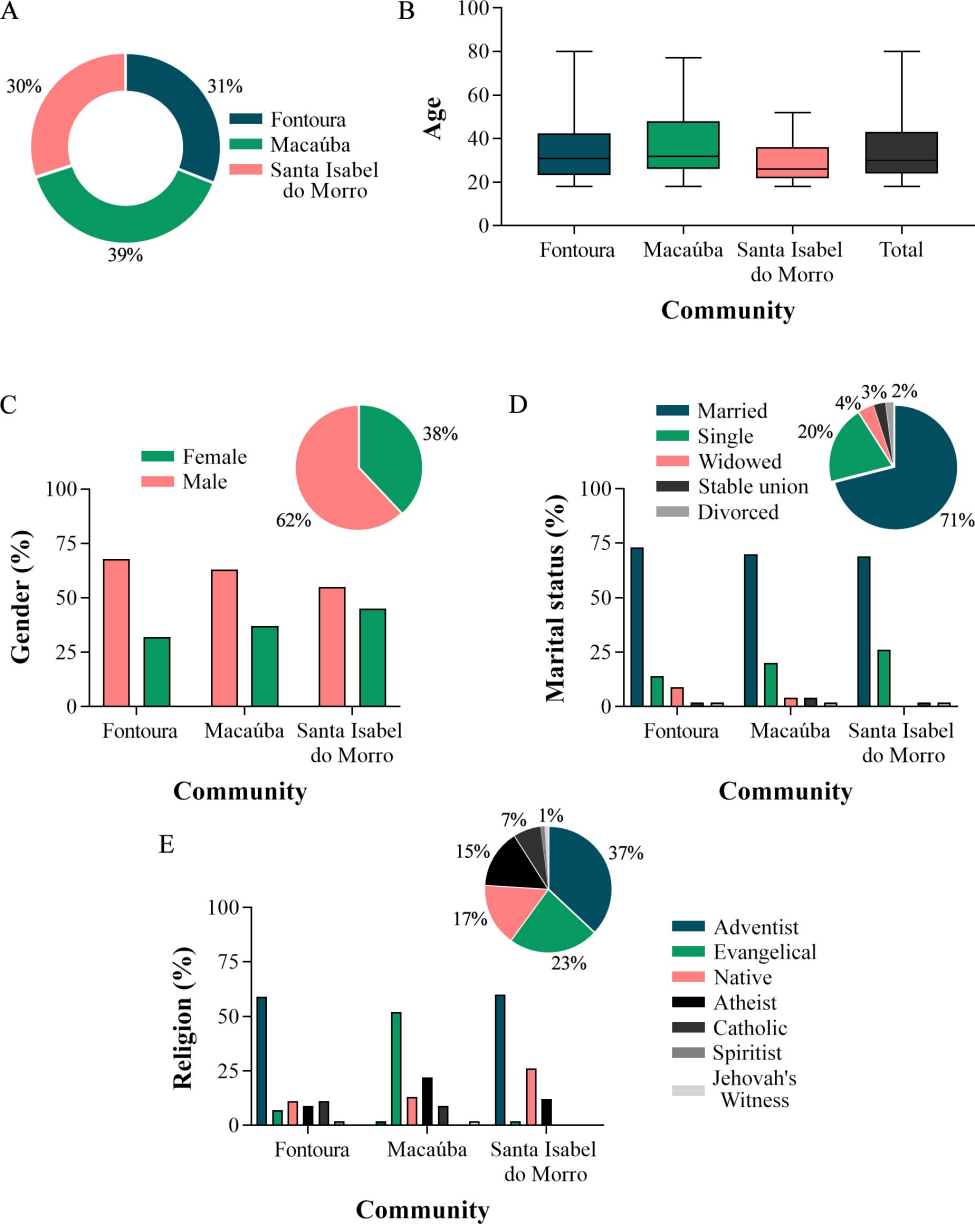
Sociodemographic data of the Karajá indigenous people from the Fontoura, Macaúba and Santa Isabel do Morro communities. (A) Percentage of interviews from each community. (B) Variation in the age of respondents. (C) Genders. (D) Marital status. (E) Religion. The bar graph represents the percentage per community and the sector graph represents the total between communities.

We interviewed 140 people, 39% from the Macaúba community, 31% from Fontoura and 30% from Santa Isabel do Morro. The average age of those interviewed was 33.9 years (standard deviation = 13.5; minimum = 18; maximum = 80 years), with 62% being male. Most interviewees stated that they were married (71%) and adhered to the Adventist religion (37%) (Fig 2).

### Data analysis

The collected data were organized and tabulated in a Microsoft Excel^©^ spreadsheet following the categorization of responses proposed by Bardin [26] and Cunha [10], and submitted to descriptive statistics, such as frequency and percentage. We used the chi-squared test to compare the number of recognized species (0 to 21) in the image album between communities and the genders of individuals. Furthermore, a Principal Component Analysis (PCA) was conducted based on a presence and absence matrix, aiming to compare the scores obtained in image recognition by each community, and to verify the relationship between the morphological groups of fungi presented in the images. We emphasize that the image album was solely used as a tool to support and represent fungal species with wide distribution in the Cerrado. During data analysis, we chose to consider the morphological group of the species represented (for example, bracket fungi, gasteroid, mushrooms, phytopathogenic fungi, among others), to avoid biased interpretations. The GraphPad Prism version 9.0.0 program (GraphPad Software, San Diego, CA, USA, www.graphpad.com) was used to create the percentage graphs, while the PCA was calculated using the *prcomp* function of the stats package. All analyzes were performed using the RStudio version 1.2.1335 program [27].

## Results

**Fig 3:**
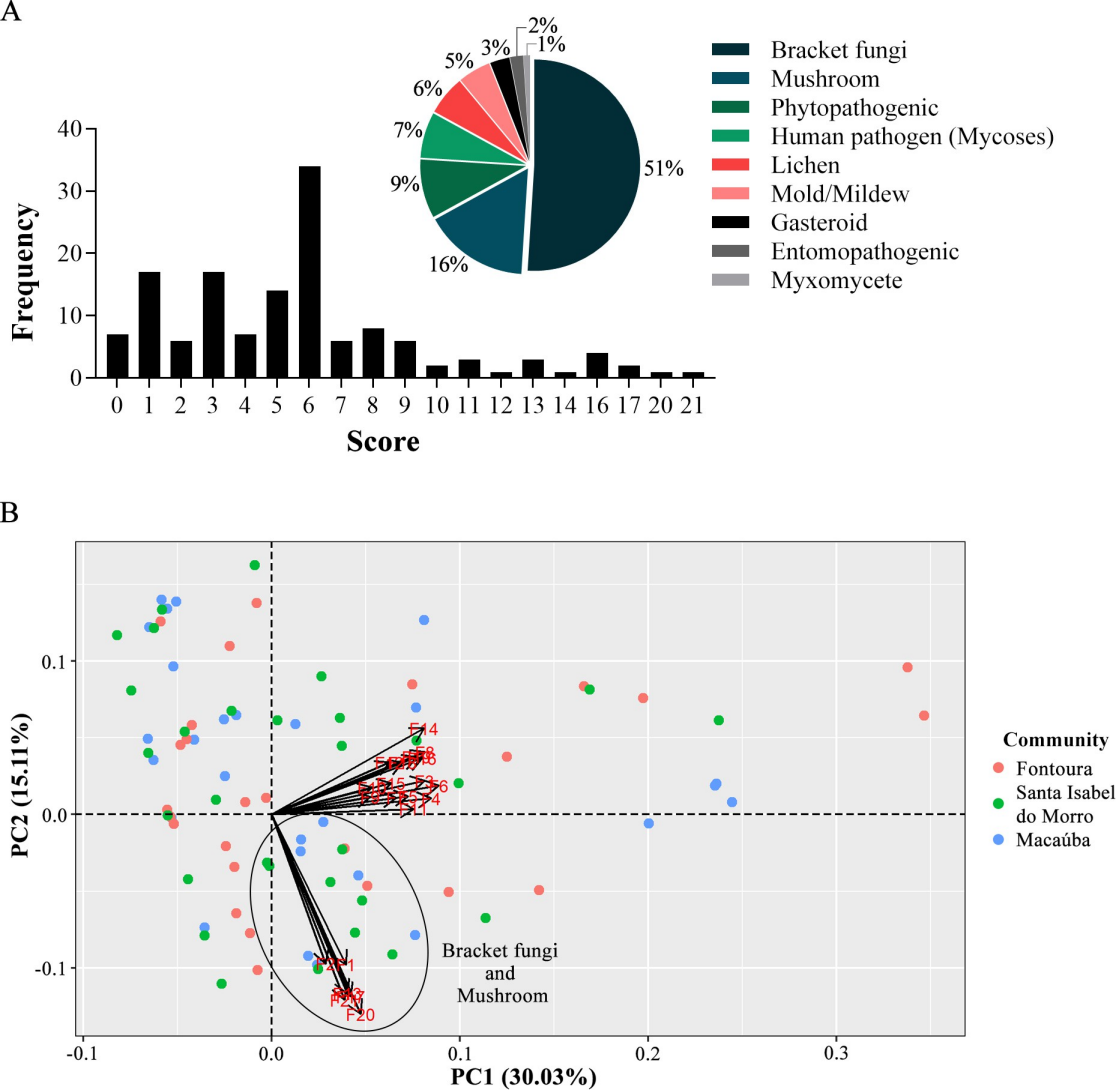
(A) Scores obtained through the recognition of species in the image album and most frequent morphological groups. (B) PCA using each respondent’s score from the three participating communities.

In this study, we observed that the Karajá people recognize the fungi of the environment in which they live, identifying some species/morphological groups in the presented image album. When categorizing the number of recognized species per interviewee, 48% recognized between 0 and 5 images, followed by 40% between 6 and 10, 6% between 11 and 15, and 6% above 15 (Fig 3).

The most frequently recognized images were those representing typical basidiomata of macrofungal species, popularly known as bracket fungi (wood ears) (51%) and mushrooms (16%), in addition to those illustrating phytopathogenic fungi (9%) and skin mycoses (7%). This relationship was also reflected in the PCA analysis, in which two groups were identified: one bringing together images of mushroom and bracket fungi species, and the other the other morphological groups of the fungi presented (Fig 3). Furthermore, the analysis revealed that the communities were not grouped by recognition of the images provided during the interview, meaning there is no significant difference between the scores obtained in this analysis and the communities (X^2^ = 3.3367, df = 2, p = 0.1886) or between the gender of the interviewees (X^2^ = 0.032981, df = 1, p = 0.8559).

**Fig 4:**
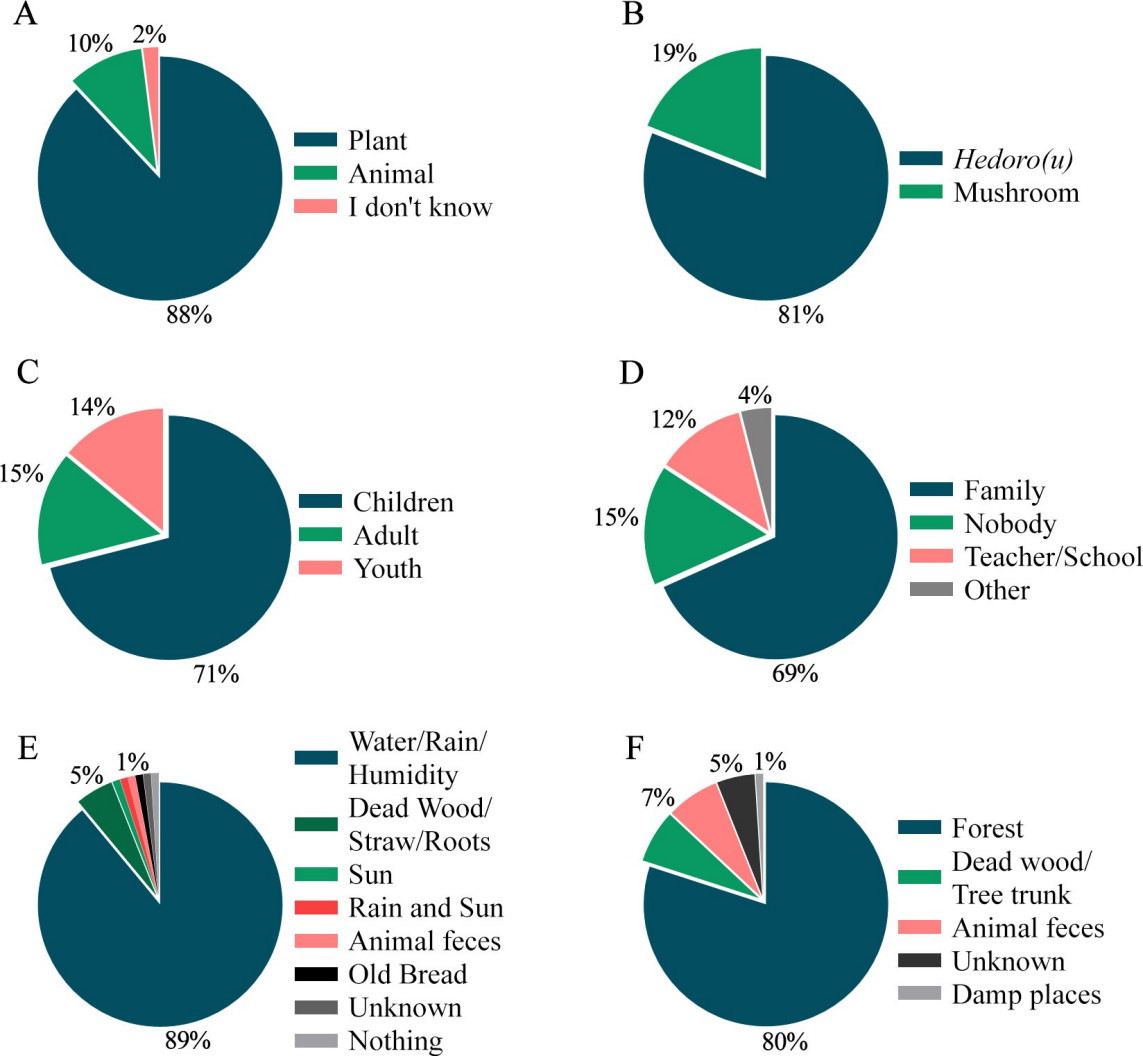
Knowledge about fungi among indigenous people of the Karajá ethnic group from the Fontoura, Macaúba and Santa Isabel do Morro communities. (A) How do you recognize these organisms? (B) What do you call these organisms? (C) How long have you known these organisms? (D) Who did you learn about these organisms from? (E) What do they need to grow? (F) Where can we find these organisms?

Although the majority of interviewees stated that they knew at least one species of fungus in the image album, none of them recognized them as fungi, but rather as plants (88%) and animals (10%) (Fig 4). They call the species found in the region *hedoro*(*u*) (81%) or mushroom (19%).

Communities have known these organisms since they were children (71%), and mainly learned about them through family knowledge (69%). Furthermore, 89% stated that fungi need water to grow, and they are mostly found in forest areas (80%), living or dead wood (7%), and animal feces (7%) (Fig 4).

**Fig 5:**
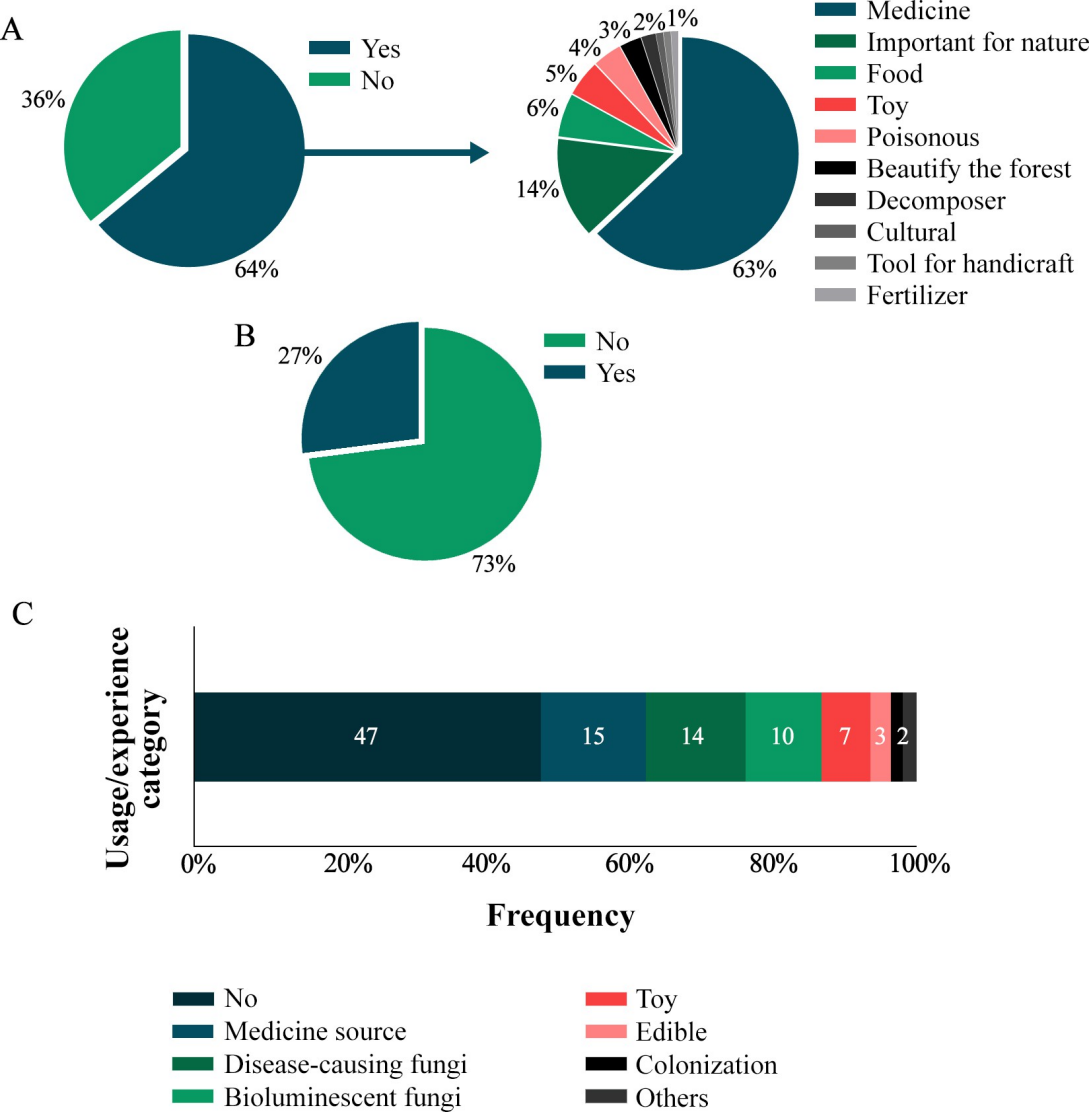
Importance and use of fungi among indigenous people of the Karajá ethnic group from the Fontoura, Macaúba and Santa Isabel do Morro communities based on the questions: (A) Are some of these organisms important? If yes, why? (B) Do you use it or know someone who uses it/has used it? (C) Usage or experience category.

When asked whether they consider fungi important, 64% of respondents responded positively. Of these, 63% attributed the importance as a source of medicine, 14% associated the importance of fungi with nature and 6% as food (Fig 5). Furthermore, 27% of people stated that they use/used or know someone who uses/used a fungus.

Personal stories or experiences related to fungi revealed that 47% of the interviewees had no such experiences. However, 52% provided positive responses, which were classified into seven distinct categories: fungi as a source of medicine, disease-causing fungi, bioluminescent fungi, use of fungi as toys or in a playful way, edible fungi, substrate colonizers and others (used in rituals, poisonous or as a tool for crafts) (Fig 5). Table 2 presents the main reports among the Karajá people related to the categories mentioned.

**Table 2:** Usage and experience reports related to fungi among indigenous people of the Karajá ethnic group from the Fontoura, Macaúba and Santa Isabel do Morro communities. The table is organized into usage/experience categories and related morphological group (when mentioned).

Morphological group	Usage/experience category
	Fungi as a medicine source
Not mentioned	“The former shaman used some to make medicines”
Mushroom and bracket fungi	“They serve as medicine”
	“Can be used against itching and fever”
	“My grandmother said it was used as a medicine for sore throats, headaches and stomachaches.”
	“My grandparents made medicine for coughs and swelling”
	“It’s good for making medicine for inflammation”
	“It can be used to make a home remedy for weakness by mixing it with alcohol”
Lichen	“Causes allergies”
Earth star (Gasteroid)	“I use it to make medicine to prevent chickens from getting sick, against gogo”
**Morphological group**	**Disease-causing fungi**
	**In plants**
Mushroom, phytopathogenic, lichen and bracket fungi	“It sucks the strength out of plants and kills the plant”
	“It causes diseases in plants and can kill the plant”
	“It rots and kills the trees”
	“It rots fruit”
Mold/mildew	“Mold grows and kills the plant”
**Morphological group**	**In animals**
Mushroom	“It makes deer, pigs and cow get sick”
	“It makes domestic animals sick”
Mold/mildew	“The fruit goes bad and the animals eat them and get sick”
**Morphological group**	**In the people of the communities**
Mushroom	“My parents forbade me to take it because it was bad for my health”
Mold/mildew	“It can harm your health, give you diarrhea and a headache”
Not mentioned	“Walking barefoot causes chilblains (perniosis)”
	“I walked barefoot in the woods and caught chilblains”
	“I already had chilblains and pityriasis versicolor (white fungal skin rash)”
**Morphological group**	**Usage/experience category**
	**Bioluminescent fungi**
Mushroom and bracket fungi	“The *hedoro*(*u*) glows in the dark”
	“There is a *hedoro*(*u*) that glows in the dark and that was used as a toy when I was a child”
	“At night they shine”
	“I picked it up under coconut trees because as it shined it was used as a type of toy”
	“When I was a child I used it as an ornament that glowed in the dark”
	“It shines on termites on the side of the road and in dense forests”
**Morphological group**	**Toy**
Mushroom	“I played with it as a child”
	“I played with mushrooms”
	“My mother used to say that children used to use them to play”
	“I played with it like an umbrella”
Gasteroid	“When I was a child I played with it, but I stopped because I thought it was poisonous.”
**Morphological group**	**Edible**
Mushroom and bracket fungi	“The oldest people used it as a form of food in the middle of the forest”
Not mentioned	“In the past it was cooked”
**Morphological group**	**Colonization**
Mushroom	“Grows in capybara feces”
	“Grows in animal feces”
	“When I built my house some appeared in the straw”
Bracket fungi	“Grows on old trees”
**Morphological group**	**Others**
Mushroom and bracket fungi	“Used as earrings and hair ornaments in the transition of boys’ sexuality”
	“Decoration during festivities”

Although the Karajá reported the use of fungi as food by older members in the past, there are currently no reports of fungi being used as a food source. However, they do recognize fungi as a source of food and potential medicine. Reports include statements such as “My grandparents made medicine for coughs and swelling” and “It’s good for making medicine for inflammation”. Veterinary use was also reported, particularly for species of the genus *Geastrum* Pers., with examples such as " I use it to make medicine to prevent chickens from getting sick, against gogo". On the other hand, there is significant recognition of fungi as pathogens, affecting plants and animals, with remarks such as "It sucks the strength out of plants and kills the plant" and "The fruit goes bad and the animals eat them and get sick". Bioluminescent fungi play a cultural and recreational role, especially in childhood, being used as toys that glow in the dark. Fungi are used as ornaments and decorations during cultural transitions and festivities, highlighting their importance in Karajá practices and beliefs. Some women reported using plant leaves contaminated with rust (a phytopathogen) to sand down handicrafts produced by the community.

## Discussion

The Karajá people were able to recognize representatives of different fungal morphological groups shown in the image album. However, they have a clearer perception of typical groups such as mushrooms and bracket fungi (wood ears), revealing a low perception of other morphological groups. This result was similar among members of the three communities studied and did not vary between the genders of the interviewees.

Although the Karajá recognize some fungal morphological groups, they understand them as plants. Historically, confusion between fungi and plants occurs in different contexts [28, 29], not only restricted to indigenous communities. This association can be attributed to some superficial similarities between the two groups of organisms, especially because they occupy the same ecological niche and because of their symbiotic and/or parasitic associations. This confusion can be even more common in indigenous communities because many people have their own methods of classifying living beings that consider criteria other than scientific ones. These people may have a broader view of nature, grouping organisms into simpler categories based on perceived characteristics or cultural aspects. It is important to highlight that the current scientific community recognizes fungi as a kingdom, with biological characteristics distinct from plants [30].

*Hedoro(u)* is the term most used by the Karajá to refer to fungi. In the *Iny rybè* language, this word is used to describe the upper rounded part of the house made of straw and is used as a synonym for mushroom. They consider the morphology of the pileus to be similar to the vaulted roof of straw houses. The term can be spelled in two ways, depending on the gender of the person using it: *hedoro* is used by women, while *hedoru* is used by men.

It is common for traditional and indigenous people to associate names with fungi based on similarities with other organisms or objects. In the Kalunga quilombola community, also located in the Cerrado domains, the population assigns compound names to fungi, referring to the substrate in which they are found, such as “bracket fungi” and “nail disease”, and based on similar morphological aspects to animals and parts of them (snake, ear) or objects (hat, umbrella) [10]. Names such as cat’s tongue, coyote’s head and deer antlers have already been recorded among the Kaqchikel indigenous people in Guatemala [31] and crispy, porcupine, tapir liver and people of white skin among the Yanomami indigenous people in Brazil [6].

We observed that learning about fungi among the population began in childhood, mainly being acquired through family knowledge. This knowledge is transmitted orally from generation to generation through narratives, rituals, ceremonies and everyday practices. Furthermore, living in a rural area enables expanding environmental perception about fungi and the understanding of the biology of the group, such as the need for water and suitable substrates for their colonization and development, as well as the harm caused by fungi as causes of illnesses. For example, some of the interviewees mentioned having had chilblains and pityriasis versicolor, and associated the consumption of fruits and other foods contaminated with fungi (such as mold/mildew) with the malaise and illness of domestic animals.

Although more than half of those interviewed stated that fungi are important, especially for producing medicine and for nature, we noticed that the community does not establish a direct connection between the use and importance of the resources offered by fungi. Only a small portion mentioned their own use of fungi. For example, a resident reported that he uses fungi from the *Geastrum* genus to treat gogo (infectious coryza) in chickens. It is interesting to highlight this report, since this genus has medicinal properties, such as antibacterial and anti-inflammatory activities [32, 33]. Furthermore, the use of gasteroid fungi has been previously reported in treating skin diseases in some countries such as Colombia [34], China [35] and India [36]. Extracts from mature fruiting bodies of these fungi have therapeutic properties and the spores can absorb water from the blood, accelerating clotting and the healing process [37].

Knowledge and stories related to fungi for most of the interviewees are linked to childhood experiences or tales told by their parents and grandparents. Many mentioned using fungi as toys or in playful activities during childhood, especially the bioluminescent fungi found associated with coconut trees and termite mounds. However, it should be noted that there are no records of bioluminescent fungi associated with termite mounds; the glowing organisms observed were likely firefly larvae with similar luminescent characteristics. Interviewees reported collecting the glowing fungi to enjoy their glow at night. The species in question is probably *Neonothopanus gardneri* (Berk.) Capelari, Desjardin, Perry, Asai & Stevani, which occurs in the Cerrado [38], and has also reported by the Kalunga quilombola community for recreational use [10].

**Fig 6:**
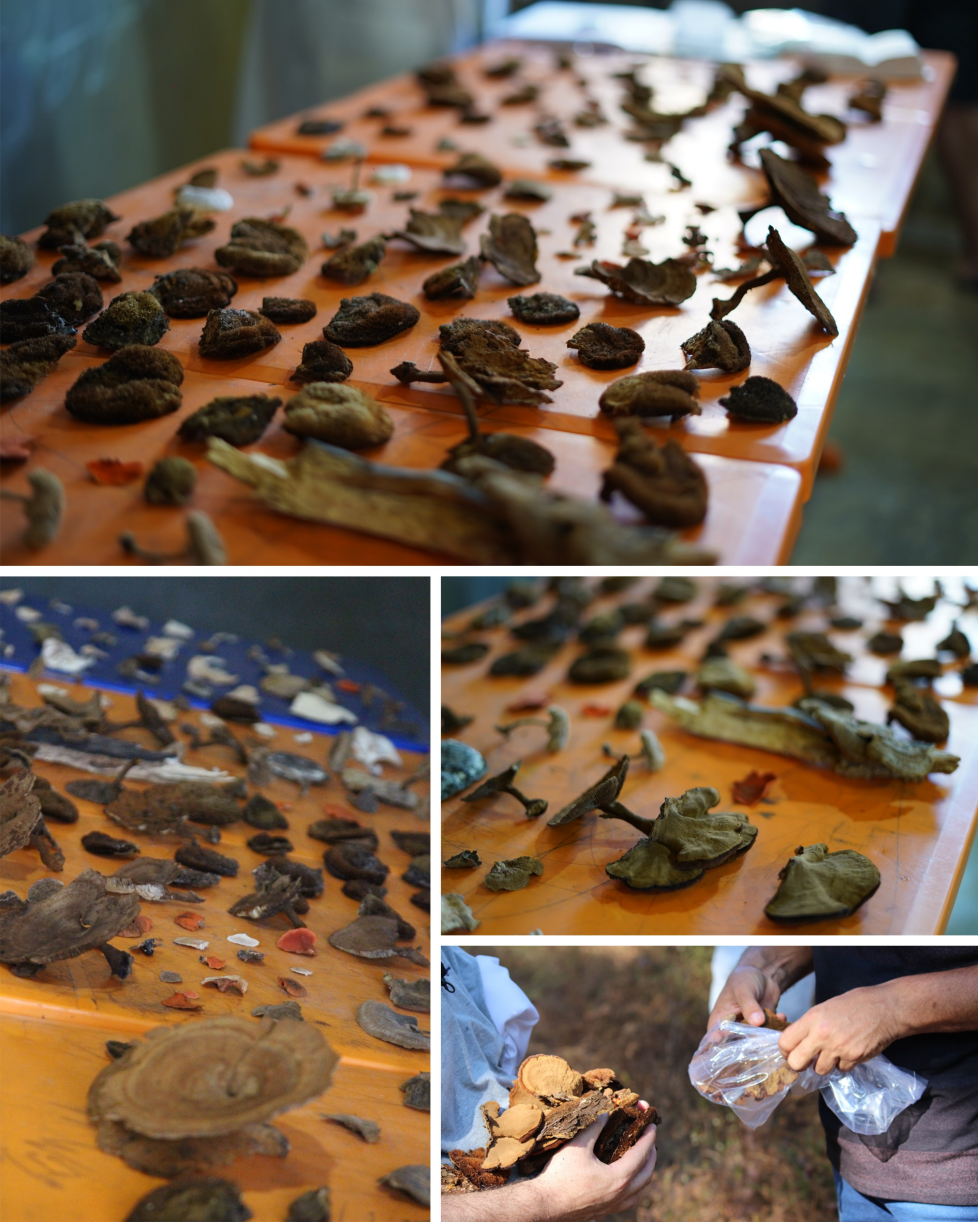
Some specimens of bracket fungi collected within the studied communities.

It is common to use mushrooms and bracket fungi as decorations in rituals and commemorative parties. During these events, especially women use these fungi as clothing items and adornments, such as earrings and hair decorations, in community rituals including Hetohoky (a male initiation rite) and the Aruanã Festival. Some of the interviewees showed some species that are used for this purpose during data collection (Fig 6).

The use of fungi in a playful way is common among children and adolescents in indigenous communities. Children of the Yuman people in Mexico play cooking games with mushrooms and use ball-of-the-earth fungi (gasteroids) as toys. They press them to watch the spores release, make moustaches with them and enjoy watching them burst [37].

Ethnomicological studies in several communities have demonstrated the wide use of mushrooms in food [39], as well as in other practices such as medicinal use and magical rituals [37]. This close relationship with fungi in a community, especially as food, is called mycophilia. However, a certain aversion to fungi has developed in some, considering them poisonous or at least indigestible, and this aversion is called mycophobia [40]. Although this classification is common, ethnomycological studies show that these concepts are influenced by ecological and sociocultural factors such as occupation, ethnicity, gender, and origin of communities [41]. These concepts should be understood as extremes of a continuum, rather than mutually exclusive categories. A community or its members can occupy an intermediate position between mycophilia and mycophobia, exhibiting attitudes that range from slightly mycophobic to highly mycophilic. Thus, although a community may not consume mushrooms in its diet, it cannot be considered mycophobic.

In the case of the Karajá, the use of fungi as a diet component was not observed, but they recognize that some species can be used for this purpose. Although there are beliefs and stories in the community that villainize fungi, especially those related to species that cause mycoses and phytopathogenic species, the Karajá are aware of the fungi around them and establish other forms of interaction with them. These interactions include use as adornments and decorations in festivities, recreational aspects, and occasionally for medicinal purposes. Therefore, we can state that the ethnicity is partially mycophilic.

The use of fungi varies from region to region and even among the same ethnic group. In countries like China, Mexico and Poland, communities have a close relationship with fungi, which are part of the local diet [37, 39, 42]. Historical record of the use of fungi by indigenous people in Brazil is relatively recent, dating back to 1965, with the publication of the study “*Conhecimento micológico dos índios brasileiros*” [43]. In this study, the author reports that Brazilian Indians did not have mycophilic habits, but were able to distinguish them from other organisms. In later studies, Fidalgo and Prance [44] and Sanuma et al. [6] mention fungi consumption by Amazonian indigenous groups, such as the Yanomami.

Traditional knowledge about a particular fungus, including its ecology, use and local name, is valuable to indigenous communities. This helps preserve their culture and strengthen their cultural identity, making the fungus (or fungi) more significant than just their practical use. Some interviewees attribute a special meaning to *hedoro*(*u*), not only because of its benefits, but also because it is linked to memories of their childhood and family. This relationship and history play an important role in maintaining and transmitting this knowledge, contributing to the sustainable conservation and use of fungi in indigenous communities.

## Final considerations

The Karajá people know fungi and have an indirect relationship with these organisms. They refer to fungi as *hedoro*(*u*), morphologically associating them with the mushroom figure and the upper, rounded part of the straw house. The concept of fungi in the community is restricted to mushrooms and morphologically similar species, such as bracket fungi. Fungi are not part of the local diet, but they are present in a playful way, especially in childhood, and are also used as a decoration in festivities, and occasionally to treat illnesses. Furthermore, we observed that fungi are frequently mentioned in relation to third parties, such as family and friends, which indicates that this knowledge is gradually being lost over time.

This study is pioneering in addressing the ethnomycological knowledge of indigenous peoples of the Cerrado and our results reinforce the need to expand research to other communities in different regions and ethnicities to expand ethnomycological knowledge among different ethnicities. These investigations could contribute to both the appreciation and conservation of the traditions and knowledge of the original peoples, as well as the biodiversity in which they are inserted.
